# Efficacy of acupuncture in children with asthma: a systematic review

**DOI:** 10.1186/s13052-015-0155-1

**Published:** 2015-07-07

**Authors:** Chi Feng Liu, Li Wei Chien

**Affiliations:** Graduate Institute of Integration of Traditional Chinese Medicine with Western Nursing, National Taipei University of Nursing and Health Sciences, No. 365 Ming-De Road, Beitou, Taipei 11211 Taiwan; Department of Obstetrics and Gynecology, Taipei Medical University Hospital, Taipei, Taiwan

**Keywords:** Acupuncture, Asthma, Child, Systematic review

## Abstract

**Background:**

We performed a systematic review of the efficacy of various types of acupuncture in the treatment of asthma in children.

**Methods:**

We searched the MEDLINE, Embase, and Cochrane Library databases up to October 20, 2014. Randomized controlled trials (RCTs) of children and adolescents (<18 years of age) with asthma were included. Data extraction was applied, and methodologic quality was assessed.

**Results:**

A total of 32 articles were assessed for eligibility, and seven studies comprising 410 patients were included in the systematic review. Two RCTs showed significant improvement in peak expiratory flow (PEF) variability for acupuncture (traditional and laser) vs. control, with one showing significant improvement in asthma-specific anxiety level, but no significant differences in other lung function parameters or quality of life. Another RCT reported significant benefits of laser acupuncture on lung function parameters but did not describe or report statistical analyses. One crossover RCT showed significant improvements in response to both acupuncture and placebo acupuncture, with better improvements with acupuncture compared to placebo acupuncture (forced exhaled volume in 1 s [FEV_1_], PEF). Two additional crossover RCTs showed no significant differences between single sessions of laser acupuncture and placebo acupuncture on baseline, postacupuncture, and postinduced bronchoconstriction values (% predicted FEV_1_, maximum expiratory flow). A recent study showed a significant effect of acupuncture paired with acupressure on medication use and symptoms in preschool-age children. Methodologic and reporting variability remains an issue. However, the results suggest that acupuncture may have a beneficial effect on PEF or PEF variability in children with asthma.

**Conclusions:**

The efficacy of acupuncture on other outcome measures is unclear. Large-scale RCTs are needed to further assess the efficacy of acupuncture in the treatment of asthma in children.

## Background

Asthma is a common inflammatory disease in children as well as adults, characterized by airway hyperresponsiveness, obstruction, mucus hyperproduction, and airway wall remodeling [1]. Worldwide, asthma is estimated to affect more than 300 million people [2], with variable prevalence of symptoms and severity in children estimated from 1 % to 37 %, depending on country [3]. The development of childhood asthma is associated with a broad range of factors including genetics, environmental factors, and lifestyle [4]. Guidelines for treatment have been published, but their implementation has been reported to be less than optimal [5]. In addition, many of the commonly prescribed drug treatments for asthma have undesirable side effects [6], including effects on growth in children by inhaled corticosteroids [7].

Acupuncture, in the form of traditional Chinese medicine with needles, as well as electroacupuncture, laser acupuncture, and transcutaneous electrical nerve stimulation, has been used for the treatment of asthma. Randomized controlled trials (RCTs) have shown efficacy of acupuncture for the treatment of allergic rhinitis, and some studies have shown positive effects of acupuncture for the treatment of asthma, atopic dermatitis, and itch [8]. In addition, acupuncture plus routine care for the treatment of patients with allergic bronchial asthma has been reported to result in additional costs but better quality of life [9]. However, several meta-analyses, including a Cochrane Review and its update, have shown no consistent evidence of a significant benefit of various forms of acupuncture in terms of efficacy [10–12]. With respect to children, a recent systematic review assessed the efficacy of laser acupuncture in the treatment of asthma, and reported no compelling evidence for the efficacy of laser acupuncture in children with asthma [13]. The objective of the present systematic review was to assess the efficacy of acupuncture in all forms on the treatment of asthma in children.

## Methods

### Search strategy, study selection, and data extraction

We searched the MEDLINE, Embase, and Cochrane Library databases up to October 20, 2014. The following search terms were used: acupuncture, electroacupuncture, transcutaneous electrical nerve stimulation, children, asthma, asthmatic. We also searched reference lists of initially identified articles. The inclusion criteria were as follows: RCTs; study subjects children and adolescents (<18 years of age) with bronchial asthma; treatment with acupuncture, electroacupuncture, laser acupuncture, or transcutaneous electrical nerve stimulation versus sham (placebo, defined as acupoint[s] not considered related to asthma or no laser light emitted) acupuncture or no acupuncture; and reporting of quantitative outcomes of interest. Studies assessing acupuncture combined with other Chinese medicine and those of children with allergic conditions other than bronchial asthma (eg, allergic rhinitis, atopic dermatitis) were excluded. Only English language articles were included.

### Data extraction

Data regarding patient characteristics, intervention, and outcome were extracted by two independent reviewers. A third reviewer was consulted for resolution of disagreements. Outcome measures extracted included pulmonary function and quality of life.

### Quality assessment

Quality assessment was performed with the Cochrane “assessing risk of bias” table [14]. This assessment includes six domains—random sequence generation, allocation concealment, blinding of patients and personnel, blinding of outcome assessment, incomplete outcome data, and selective reporting risk.

## Results

**Fig. 1:**
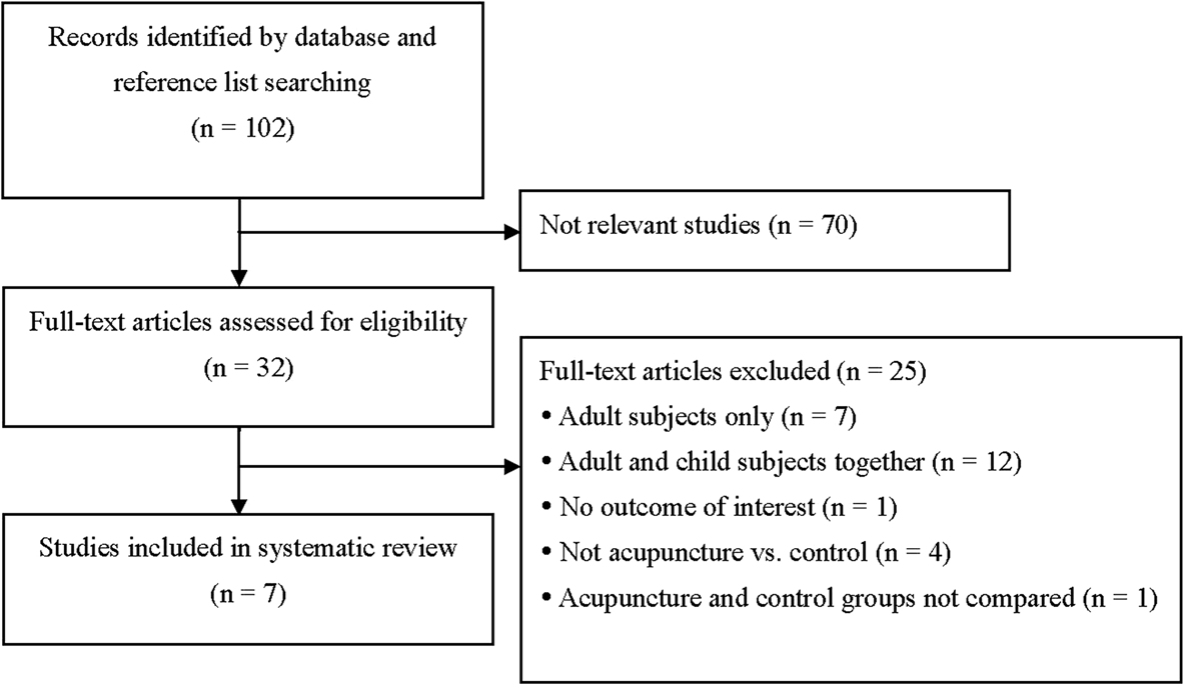
Flow diagram of study selection

The search process is detailed in Fig. 1. The search resulted in a total of 102 articles. Seventy of these articles were deemed not relevant, and 32 were assessed for eligibility. A total of 25 articles were excluded for the following reasons: performed in adults and not in children (*n* = 7), included adults as well as children (*n* = 12), not acupuncture vs. control (*n* = 4), acupuncture and control groups not compared (*n* = 1), and no outcome of interest (*n* = 1). A total of seven articles were included in the systematic review [15–21].

Characteristics of the seven RCTs, including 410 patients, are listed in Table 1. Four studies assessed traditional acupuncture [15, 16, 20, 21], and three studies assessed laser acupuncture [17–19]. The stage of asthma varied, with one study assessing intermittent or mild asthma [18], one assessing acute bronchial obstruction [17], two not specifying other than bronchial asthma [15, 16], and three assessing exercise-induced asthma [19–21]. With the exception of one study of preschool-age children [15], patient age was generally in the adolescent range (9–16 years of age), and the gender distribution was balanced in the three studies that reported gender.

**Table 1:** Characteristics of included studies

Author (year)	Study design	Patients	Stage of asthma	Number of patients	Age years (mean ± SD)	Gender M/F	Intervention	Acupoint(s)
Karlson and Bennicke (2013) [15]	RCT	Children ages 6 months to 6 years with asthma	NA	64	2.8 ± 1.6	NA	Acupuncture + acupressure: 10 sessions (1.5 h each) over a period of 3 months	Acupuncture: LIV (liver) 2
								Acupressure: LU 7, REN 17, REN 22, LI 4, PC 6, SP 6, SP 10, ST 36, ST 40, K 3, K 7, DU 14, DU 20, Dingchuan
				58	2.2 ± 1.1		Control: No acupuncture	None
Scheewe et al. (2011) [16]	RCT	Children and adolescents ages 12-17 years with bronchial asthma	NA	46	Male: 14.6 ± 1.8	26/20	Acupuncture: Total of 12 sessions (30 min each) over a 4-week period	Bilateral bladder 13; bilateral conception vessel 17; bilateral lung 7; plus 2-6 additional points
					Female: 15.0 ± 2.1			
				47	Male: 13.7 ± 1.2	25/22	Control: No acupuncture	None
					Female: 16.2 ± 1.7			
Nedeljković et al. (2008) [17]	RCT	Children ages 7-17 years with asthma	Acute bronchial obstruction	50	11	25/25	Su Jok therapy (laser at acupuncture points in hand): Total 10 sessions (5 each week except for weekends over 12 days)	According to the 6-Ki Su Jok principles
				50		25/25	Control: No acupuncture	None
Stockert et al. (2007) [18]	RCT	Children ages 6-12 years with asthma	Intermittent or mild	7	9.4 ± 2.4	NA	Laser acupuncture (10 treatments of laser acupuncture on a once-weekly schedule) + probiotic drops (20 drops 3 times daily for 7 weeks)	Lung 1, 5, 7, 9, 11; large intestine 4, 6, 19, 20; bladder 13, 17, 18, 20, 21, 23; stomach 13, 25, 36, 40, 44; spleen 3, 6, 9, 10; heart 3, 5, 7; small intestine 3; kidney 3, 6, 8, 27; pericardium 6; triple heater 5, 15; gallbladder 3, 34, 40, 41; liver 2, 3, 8, 13; Yintang, Dingchuan; conception vessel 4, 6, 9, 17, 21; governor vessel 4, 13
				9	10.0 ± 1.9		Control: 10 placebo laser acupuncture sessions + placebo drops	No laser light
Gruber et al. (2002) [19]	RCT, crossover	Children with exercise-induced asthma	Mild to moderate	44	11.9 ± 2.6	26/18	Laser acupuncture: Single treatment	Yintang (EX 1), Chize (LU 5), Lieque (LU 7), Feishu (UB 13), Geshu (UB 17), and Shanzhong (CV 17)
							Control: Laser acupuncture at placebo points; single treatment	Baihui (GV 20), Quze (PE 3), Neiguan (PE 6), Dashu (UB 11), Weishu (UB 21), and Zhongwan (CV 12)
Fung et al. (1986) [20]	RCT, crossover	Children with exercise-induced asthma	Mild to moderate	19	Range 9-13.5	NA	Acupuncture: Applied 20 min before exercise	Dingchuan (extra 17), kongzui (L6), and taixi (K3)
						NA	Control: No acupuncture or acupuncture applied at placebo points 20 min before exercise	Placebo points: Jianwaishu (SI14), ximen (P4), and xuanzhong (G39)
Chow et al. (1983) [21]	RCT, crossover	Children with exercise-induced asthma	NA	16	11 (range 8-13)	NA	Acupuncture: Applied 10 min before exercise (once per week for 2 weeks)	Auricular lung point
							Control: No acupuncture (baseline) or acupuncture applied at placebo point	Auricular lumbago point

F, female; M, male; NA, not available/applicable; RCT, randomized controlled trial; SD, standard deviation

Lung parameter outcomes for were reported in six of the seven RCTs (Table 2), and medication use, symptoms, and quality of life outcomes were reported in three of the seven studies (Table 3). The most recent study, by Karlson and Bennicke [15], assessed the effect of 10 sessions of acupuncture plus ongoing acupressure treatment over a period of 3 months in preschool-age children with asthma (the youngest age group among the studies). The outcome measures of medication use (inhaled steroids [IHS]) and symptom reduction were measured via diary. The results showed a statistically significant reduction of symptoms (*P* = 0.0376) and IHS use (*P* = 0.0005) with acupuncture/acupressure compared to no acupuncture/acupressure at 3 months; however, this effect was not sustained at a 12-month follow-up (Table 3).

**Table 2:** Outcomes for lung function parameters (data reported for six of seven studies)

Author (year)	Intervention	FEV_1_			FVC			PEF			MEF		
		Mean ± SD, or %			Mean ± SD, or %			Mean ± SD, or %			Mean ± SD, or %		
		Before	After	Change	Before	After	Change	Before	After	Change	Before	After	Change
Scheewe et al. (2011) [16]	Acupuncture	–	–	–	FEV_1_/FVC	FEV_1_/FVC	–	PEF variability 16.1 % ± 8.3 %	PEF variability 12.5 % ± 5.5 % *	–	MEF_50_ 88.5 % ± 26.8 %	MEF_50_ 89.2 % ± 26.1 %	–
					84.6 ± 9.0	85.4 ± 8.6							
	No acupuncture	–	–	–	FEV_1_/FVC	FEV_1_/FVC	–	PEF variability 20.4 % ± 9.4 %	PEF variability 16.9 % ± 8.8 %	–	MEF_50_ 86.8 % ± 27.9 %	MEF_50_ 86.2 % ± 23.1 %	–
					84.6 ± 10.1	84.9 ± 8.3							
Nedeljković et al. (2008) [17]†	Su Jok therapy (laser)	51 %	84 % *	–	62 %	90 % *	–	65 %	96 % *	–	–	–	–
	No acupuncture	58 %	74 %	–	70 %	86 %	–	64 %	78 %	–	–	–	–
Stockert et al. (2007) [18]	Laser acupuncture	81.8 % ± 8.1 %	86.3 % ± 5.7 %	–	–	–	–	PEF variability 25.2 % ± 16.2 %	PEF variability 7.9 % ± 2.9 % *	–	–	–	–
	Placebo acupuncture	81.5 % ± 8.0 %	88.6 % ± 8.5 %	–	–	–	–	PEF variability 15.5 % ± 1.8 %	PEF variability 17.8 % ± 23.9 %	–	–	–	–
Gruber et al. (2002) [19]‡	Laser acupuncture	98.5 % ± 9.8 %	84.2 % ± 15.3 %	14.9 % ± 14.1 %§	–	–	–	–	–	–	MEF_25_ 78.6 % ± 23.1 %	MEF_25_ 56.3 % ± 24.5 %	MEF_25_ 29.7 % ± 23.2 % §
	Placebo acupuncture	97.8 % ± 11.0 %	86.2 % ± 12.6 %	12.4 % ± 10.2 %§	–	–	–	–	–	–	MEF_25_ 78.4 % ± 26.1 %	MEF_25_ 57.3 % ± 23.1 %	MEF25 28.9 % ± 21.3 % §
Fung et al. (1986) [20]	Acupuncture	–	–	23.78 % ± 19.46 % *¶	–	–	15.80 % ± 20.71 % ¶	–	–	25.90 ± 20.76 % *¶	–	–	–
	Placebo acupuncture	–	–	32.63 % ± 19.99 % *¶	–	–	26.13 % ± 17.76 % ¶	–	–	34.34 ± 18.97 % *¶	–	–	–
	No acupuncture	–	–	44.38 % ± 16.88 % ¶	–	–	33.25 % ± 21.37 % ¶	–	–	49.54 % ± 17.25 % ¶	–	–	–
Chow et al. (1983) [21]	Acupuncture	1.53 L (SEM: 0.09 L)	1.14 L (SEM: 0.12 L)	27.5 % (SEM: 5.36 % ¶)	–	–	–	–	–	–	–	–	–
	Placebo acupuncture	1.50 L (SEM: 0.07 L)	1.02 L (SEM: 0.10 L)	31.9 % (SEM: 7.98 % ¶)	–	–	–	–	–	–	–	–	–
	No acupuncture	1.55 L (SEM: 0.07 L)	0.92 L (SEM: 0.07 L)	40.2 % (SEM: 10.0 % ¶)	–	–	–	–	–	–	–	–	–

*,Significant difference between groups

†, Data from this study are estimated from graphically presented values

‡, Data from this study are presented as % predicted values

§, Percent fall postacupuncture to 15 min after cold dry air challenge

¶, Attenuation of exercise-induced asthma as measured by percent fall in FEV_1_, FVC, or PEF

–, Not reported

FEV_1_, forced exhaled volume in 1 s; FVC, forced vital capacity; MEF, maximum expiratory flow (at 50 % or 25 % of the FVC); PEF, peak expiratory flow; SD, standard deviation

**Table 3:** Outcomes for medication use, symptoms, and quality of life (data reported for three of seven studies)

Author (year)	Intervention	Medication use			Symptoms			Quality of life			
								Mean ± SD			
		Before	After	Follow-up	Before	After	Follow-up	Before	After	Follow-up	Scale used
Karlson and Bennicke (2013) [15]	Acupuncture	β-mimetics (puffs/24 h) 2.2 ± 2.1	β-mimetics (puffs/24 h) 0.7 ± 1.1	β-mimetics (puffs/24 h) 0.4 ± 1.0	*Significant reduction in symptoms between groups at 3 months; however, no significant difference at 12 months			–	–	–	–
		IHS (μg/day)537 ± 260	IHS (μg/day) 126 ± 209*								
	No acupuncture	β-mimetics (puffs/24 h) 2.8 ± 4.2	β-mimetics (puffs/24 h) 0.9 ± 1.3	β-mimetics (puffs/24 h) 0.5 ± 1.0				–	–	–	–
		IHS (μg/day) 477 ± 271	IHS (μg/day) 342 ± 287								
Scheewe et al. (2011) [16]	Acupuncture	Controller (frequency/ day) 1 use: 23	Controller (frequency/ day) 1 use: 9	Controller (frequency/ day) 1 use: 4	(Per day) Morning: 18 Day: 19	(Per day) Morning: 13 Day: 13	(Per day) Morning: 18 Day: 18	Overall: 3.8 ± 0.7	Overall: 4.3 ± 0.5	Overall: 4.2 ± 0.6	PAQLQ
								Activity: 3.2 ± 0.9	Activity: 4.0 ± 0.8	Activity: 3.9 ± 1.0	
								Symptom: 3.5 ± 0.9	Symptom: 4.2 ± 0.7	Symptom: 3.9 ± 0.9	
		≧2 uses: 18	≧2 uses: 25	≧2 uses: 36	Night: 10	Night: 7	Night: 9	Emotion: 3.8 ± 1.0	Emotion: 4.5 ± 0.6	Emotion: 4.3 ± 0.9	
								Trait: 32.4 ± 5.8	Trait: 32.5 ± 8.2	Trait: 29.1 ± 7.3	STAIC
								State: 41.5 ± 8.1	State: 38.4 ± 8.7*	State: 37.6 ± 8.2	
	No acupuncture	Controller (frequency/day) 1 use: 30	Controller (frequency/ day) 1 use: 9	Controller (frequency/ day) 1 use: 6	(Per day) Morning: 13 Day: 11	(Per day) Morning: 11 Day: 14	(Per day) Morning: 18 Day: 15	Overall: 3.4 ± 0.8	Overall: 4.1 ± 0.6	Overall: 3.9 ± 0.9	PAQLQ
								Activity: 3.2 ± 0.8	Activity: 4.2 ± 0.7	Activity: 4.0 ± 0.7	
								Symptom: 3.7 ± 0.8	Symptom: 4.2 ± 0.6	Symptom: 4.1 ± 0.8	
		≧2 uses: 13	≧2 uses: 26	≧2 uses: 34	Night: 4	Night: 4	Night: 9	Emotion: 4.0 ± 0.9	Emotion: 4.5 ± 0.6	Emotion: 4.3 ± 0.7	
								Trait: 36.4 ± 8.9	Trait: 33.6 ± 10.0	Trait: 30.4 ± 9.5	STAIC
								State: 45.2 ± 8.8	State: 44.4 ± 9.2	State: 42.5 ± 10.3	
Stockert et al. (2007) [18]	Laser acupuncture	β-mimetics (puffs/week)	β-mimetics (puffs/week)	–	–	–	–	62.1 ± 20	54.9 ± 25.4	49.3 ± 23.8	ACQ
		8.7 ± 15.4	9.1 ± 15.4								
	Placebo acupuncture	β-mimetics (puffs/week)	β-mimetics (puffs/week)	–	–	–	–	47.8 ± 9.5	34.6 ± 16.6	38.7 ± 17.1	
		3.1 ± 6.2	3.7 ± 6.7								

*,Significant difference between groups

–, Not reported

IHS, inhaled steroids; PAQLQ, Paediatric Asthma Quality of Life Questionnaire; STAIC, State-trait anxiety inventory for children; ACQ, Asthma Control Questionnaire

The study by Scheewe et al. [16] assessed the effect of 12 acupuncture sessions over a period of 4 weeks on bronchial asthma in the inpatient setting. The control group participated in a discussion group. Patients maintained drug therapy throughout. Of the lung function parameters assessed, a statistically significant (*P* < 0.01) improvement in peak expiratory flow (PEF) variability (calculation information not included in the report) was shown in the acupuncture group vs. the control group (Table 2). No significant differences between groups were observed with respect to other lung function parameters (ratio of forced exhaled volume in 1 s [FEV_1_] to forced vital capacity [FVC]; or deltaFEV_1_ or maximum expiratory flow at 50 % of FVC [MEF_50_]) or medication use, symptoms, or quality-of-life variables (Table 3), although a significant improvement in asthma-specific anxiety level was reported.

The study by Nedeljković et al. [17] assessed the effect of laser therapy applied at acupuncture points in the hand (Su Jok therapy) for a total of 10 sessions over a period of 12 days in the outpatient setting. Subjects continued conservative drug therapy throughout the study period. Lung function parameters (FEV_1_, FVC, and PEF, Table 2; as well as forced expiratory flow [FEF], not shown in Table 2) were assessed on Days 0, 5, and 12. The text states that lung function measurements in the acupuncture group returned to normal values by Day 12, whereas those in the control group did not, and that the results obtained in the acupuncture group and control group differed with a high rate of statistical significance. However, no statistical methods are provided or results (ie, SD values, *P* values) shown. Therefore, it is not possible to determine whether the shown % changes in lung function parameters between groups or at Day 12 vs. Day 0 are indeed significant.

The study by Stockert et al. [18] assessed the effect of 10 treatments of once-weekly laser acupuncture plus probiotic drops three times daily for 7 weeks (the combined regimen referred to as Traditional Chinese Medicine) compared to placebo laser therapy plus placebo drops on intermittent or mild asthma. Of the lung function parameters assessed, a statistically significant (*P* = 0.034) improvement in PEF variability was shown in the acupuncture group vs. the placebo acupuncture group and also for acupuncture vs. baseline (*P* = 0.015) (Table 2). PEF variability was calculated as follows:

$$ \mathrm{P}\mathrm{E}\mathrm{F}\ \mathrm{variability} = \frac{\left(\mathrm{P}\mathrm{E}\mathrm{F}\ \mathrm{evening}\ \hbox{--}\ \mathrm{P}\mathrm{E}\mathrm{F}\ \mathrm{morning}\right) \times 100}{\left(\mathrm{P}\mathrm{E}\mathrm{F}\ \mathrm{evening} + \mathrm{P}\mathrm{E}\mathrm{F}\ \mathrm{morning}\right) \times 0.5} $$

No significant differences between groups were observed with respect to other lung function parameters (FEV_1_), medication use, or quality-of-life (Table 3).

The study by Gruber et al. [19] assessed the effects of single sessions of laser and placebo laser therapy applied in random order over two consecutive days (crossover design) on mild to moderate exercise-induced asthma in the outpatient setting, as measured by cold dry air hyperventilation–induced bronchoconstriction. Medications were withheld for 12 (bronchodilator) to 24 (long-term medications) hours before the study. Results showed no statistically significant differences between the laser acupuncture and placebo acupuncture groups in baseline (control), postacupuncture, and 3- and 15-min post-induced bronchoconstriction values (% predicted FEV_1_ as well as % predicted MEF_25_).

The study by Fung et al. [20] assessed the effects of acupuncture and placebo acupuncture on mild to moderate exercise-induced asthma in the outpatient setting. A first weekly session was performed without any acupuncture (control), followed by two weekly sessions of acupuncture or placebo acupuncture in random order applied 20 min before exercise (crossover design). The subjects refrained from the use of any medication 24 h before the study day. Both acupuncture and placebo acupuncture showed statistically significant improvements in lung function parameters (difference in mean percentage fall in FEV_1_, FVC, and PEF) compared to control (*P* < 0.02 by two-way ANOVA) (Table 2). However, pairwise comparisons showed better improvements in response to acupuncture compared to placebo acupuncture (FEV_1_, *P* < 0.01; PEF, *P* < 0.05). The statistical difference in mean percentage fall in FVC between acupuncture and placebo acupuncture groups was not reported.

The study by Chow et al. [21] assessed the effects of acupuncture and placebo acupuncture on exercise-induced asthma. A first weekly session was performed without any acupuncture (control), followed by two weekly sessions of acupuncture or placebo acupuncture in random order applied 10 min before exercise (crossover design). The subjects refrained from the use of medications for 8-24 h before testing. The results showed no significant differences in FEV_1_ between groups (Table 2).

Results of quality assessment are shown in Table 4. All seven studies were RCTs, but the details of randomization were not detailed. One acupuncture study [20] and two laser acupuncture studies [18, 19] were double-blind (patient and technician assessing outcomes) with respect to the type of acupuncture (acupuncture or placebo) performed. One additional study [21] was single-blind (patients were blind to the type of acupuncture performed).

**Table 4:** Quality assessment of included studies

Author (year)	Random sequence generation (selection bias)	Allocation concealment (selection bias)	Blinding of participants and personnel (performance bias)	Blinding of outcome assessment (detection bias)	Incomplete outcome data (attrition bias)	Selective reporting (reporting bias)	Did the analysis include an intent-to-treat analysis?
Karlson and Bennicke (2013) [15]	Y	NA	N	N	Y	Y	NA
Scheewe et al. (2011) [16]	Y	NA	N	NA	Y	Y	NA
Nedeljković et al. (2008) [17]	Y	NA	N	NA	NA	Y	NA
Stockert et al. (2007) [18]	Y	Y	Y	Y	Y	Y	N
Gruber et al. (2002) [19]	Y	NA	Y	Y	NA	Y	NA
Fung et al. (1986) [20]	Y	NA	Y	N	NA	Y	NA
Chow et al. (1983) [21]	Y	NA	Y	N	NA	Y	NA

NA, not available; N, no; Y, yes

## Discussion

The aim of this review was to assess published reports regarding the efficacy of acupuncture for the treatment of asthma in children. The original aim was to perform an updated meta-analysis; however, the quality and presentation of the data in the seven articles were insufficient for statistical comparison. Therefore, we performed a systematic review. The results indicate that acupuncture, in the form of traditional needle acupuncture or laser acupuncture, may provide benefit with respect to PEF and perhaps FEV_1_, along with asthma-specific anxiety level. However, these findings are based on the results of three studies and should be interpreted with caution. In addition, the results of Karlson and Bennicke [15] indicate a significant effect of acupuncture on medication use and symptoms in younger children. The quality assessment shows that the study quality was not high, with four of the studies being blinded. In addition, the acupuncture points used varied among studies.

The presentation of the outcome measures assessed in the seven studies makes it difficult to directly compare results. Whereas six of the seven studies assessed objective lung function (FEV_1_, FVC, PEF), the presentation as PEF variability, FEV_1_/FVC, % fall, % predicted value, and presentation in graphic vs. numeric form prevented direct comparison. Other outcome measures, such as quality of life, anxiety, and respiratory symptoms assessed via visual analog scale, were not consistently reported.

In addition to variable outcome measure presentation, heterogeneity in the acupuncture methods used (type of acupuncture, application placement, depth, time, number of sessions), experimental conditions (induced vs. noninduced asthma), testing environment (inpatient vs. outpatient), and controls (no treatment vs. placebo), may alter conditions sufficiently to mask any real effect of acupuncture in this context. For example, the single sessions used in the study by Gruber et al. [19] may not have been sufficient to elicit an effect. In addition, three of the studies in the present analysis [19–21] were excluded from a Cochrane review because the asthma assessed was induced [10]. Moreover, the significant but limited beneficial effects reported by Scheewe et al. [16] were obtained in the inpatient environment; the authors suggest that stronger effects might be obtained in the outpatient environment. Finally, the finding of significant effects in response to placebo acupuncture (but less so than in response to acupuncture) in the study by Fung et al. [20] suggest the possibility of a placebo effect, but there is concern that the placebo points used may not be strictly placebo (ie, they may be relevant to asthma) [10], as well as concern regarding the relevance of the acupuncture points used to treat asthma. It should also be considered that acupuncture might indirectly affect allergic diseases by modulating the production of inflammatory cytokines [22].

## Conclusions

Methodologic variability remains an issue. However, the results suggest that acupuncture may have a beneficial effect with respect to PEF or PEF variability in children with asthma. The efficacy of acupuncture on other outcome measures is unclear. Along with the issues discussed above, limitations of the present review include the small number of included studies, with relatively few patients. Large-scale RCTs using similar methodology and assessing similar outcome measures are needed to further assess the efficacy of acupuncture in the treatment of children with asthma.

## Abbreviations

FEV: Forced exhaled volume
FVC: Forced vital capacity
PEF: Peak expiratory flow
